# Severe hyperkalemia is prevalent and can be detected by quantitative electrocardiographic findings and medication history of calcium channel blocker among the patients with symptomatic or extreme bradycardia

**DOI:** 10.1186/cc12234

**Published:** 2013-03-19

**Authors:** SB Chon

**Affiliations:** 1 Kangwon National University Hospital, Chuncheon, South Korea

## Introduction

Severe hyperkalemia (serum potassium ≥6.0 mmol/l with electrocardiography (ECG) change) should be detected and treated immediately [1,2]. Among symptomatic or extreme bradycardia patients, we sought its prevalence and prediction rule by history, vital sign, and ECG [3,4].

## Methods

A retrospective cross-sectional study was performed on patients with symptomatic (heart rate (HR) ≤50/minute with dyspnea, chest pain, altered mentality, dizziness/syncope/presyncope, general weakness, oliguria, or shock) or extreme (HR ≤40/minute) bradycardia at an ED of an academic hospital from June 2008 to March 2012. Risk factors of severe hyperkalemia were chosen by multiple logistic regression analysis among history (gender, age, comorbidity, and current medication), vital sign, and ECG (maximal precordial T wave amplitude, PR and QRS intervals). Scoring index was derived by summing up of simplified regression coefficients of independent risk factors.

## Results

A total of 169 cases were enrolled. Mean age was 71.2 years (SD, 12.5 years). Females numbered 87 (51.5%). Thirty-six cases (21.3%) had severe hyperkalemia. Four variables were independent risk factors of severe hyperkalemia (simplified scores in parentheses): medication of calcium channel blocker (CCB: 2); maximal precordial T ≥8.5 mV (2); PR (atrial fibrillation or junctional bradycardia: 1); and HR ≤40/minute (1). (Nagelkerke *R*^2 ^= 0.503, AUC = 0.849 (95% CI 0.786 to 0.899).) Sensitivity and specificity reached 0.75 and 0.83 when total score was ≥3. For score ≥4, positive likelihood ratio reached 5.54 (sensitivity 0.50, specificity 0.91).

## Conclusion

Severe hyperkalemia is prevalent among symptomatic or extreme bradycardia patients and could be detected immediately by a scoring index composed of quantitative ECG parameters and history of medication of CCB.
